# Human Papillomavirus (HPV) Infection and Its Impact on Male Infertility

**DOI:** 10.3390/life12111919

**Published:** 2022-11-18

**Authors:** Giuseppina Capra, Tiziana Notari, Michela Buttà, Nicola Serra, Giovanni Rizzo, Liana Bosco

**Affiliations:** 1 UOC of Microbiology and Virology, Polyclinic Hospital, Via del Vespro, 133, 90127 Palermo, Italy; 2 Department of Health Promotion, Mother and Child Care, Internal Medicine and Medical Specialties (ProMISE) “G. D’Alessandro”, University of Palermo, Piazza delle Cliniche, 2, 90127 Palermo, Italy; 3 “Check Up” Polydiagnostic and Research Laboratory, Andrology Unit, Viale Andrea De Luca 5, 84131 Salerno, Italy; 4 “D’Arena”—Clinical Analysis and Diagnostics Laboratory—Vallo della Lucania, Str. Giuseppe Garibaldi, 25/C-F, 84078 Salerno, Italy; 5 Department of Public Health, University Federico II of Naples, Via S. Pansini 5, 80131 Naples, Italy; 6 Department of Biomedicine, Neuroscience and Advanced Diagnostics (Bi.N.D), Section of Biology and Genetics, University of Palermo, 90133 Palermo, Italy

**Keywords:** HPV infection, DFI, SCD, differential lysis procedure

## Abstract

Nowadays, the striking numbers of infertile couples that turn to assisted reproductive technologies (ART) drive the research toward a more comprehensive understanding of the underlying causes. Male factors contribute to the inability to conceive in half of the cases, and it has been suggested that sexually transmitted infections could have a role in the onset of spermatozoa impairments. Since the impact of HPV infection on sperm quality and sperm DNA integrity is debated, we wanted to analyze its impact on conventional seminal parameters and the sperm DNA fragmentation index (DFI). Therefore, 117 semen samples of patients undergoing IVF were evaluated for the following characteristics: HPV DNA detection and sperm DNA fragmentation, concentration, motility, and morphology. The results showed a higher rate of HPV-negative patients (59.8% vs. 40.2%) and no HPV-related effect on DFI, sperm concentration, total sperm number, and total motility. Only progressive motility and morphology were found as significantly influenced by HPV positivity. Moreover, we observed a statistically significant difference in DFI when comparing high-risk HPV (HR-HPV) and low-risk HPV (LR-HPV) genotypes. Our data suggest that the presence of any HPV type, whatever the exact localization of the virions, can impair some sperm parameters, while HR-HPVs specifically affect the integrity of spermatozoa DNA.

## 1. Introduction

Infertility, defined as the inability to conceive after 12–24 months of unprotected sexual intercourse [1], is recognized as a rising issue, affecting almost 15% of reproductive-aged couples worldwide [2]. When searching for the roots of this common problem, male factors are indicated as the primary cause in about 30% of cases and as a contributing factor in half of infertile couples [3,4]. 

The etiology of male infertility is complex; to understand its causes, routine laboratory practice involves the basic examination of spermatozoa parameters, such as count, concentration, motility, and morphology [5] (pp. 21–102). However, this conventional analysis is often insufficient to distinguish fertile from infertile males, and it needs to be combined with the evaluation of more reliable markers, including sperm genomic integrity [4]. 

Damage of spermatozoa DNA, measured as the DNA fragmentation index (DFI), is a crucial factor negatively affecting embryo development, implantation, and pregnancies in spontaneous and assisted reproduction [6]. Studies have shown a high prevalence of DFI both in men with abnormal ejaculate parameters and normozoospermic individuals [7] (pp. 87–100). 

The reasons behind spermatozoa genomic impairments could be several, from abnormalities in DNA packaging during spermatogenesis to aberrant apoptosis and exposition to oxidative stress. Environmental and pathological conditions, such as diseases, drugs, aging, and infections, are the main triggers [7] (pp. 75–81).

Among the sexually transmitted diseases, human papillomaviruses (HPVs) infection is the most widespread: it is estimated that about 80% of sexually active men and women will contract the virus at least once over their lifetime [8]. 

More than 200 different HPV genotypes have been described to date, all characterized by a high tropism for skin and mucous membranes’ epithelia [9]. Most of the time, infections are cleared by the immune system in the space of 12–24 months, thus avoiding any clinical outcomes. However, failure to eliminate the virus leads to the establishment of a persistent infection, which is believed to be the necessary cause for the development of neoplastic hyperproliferative lesions [10]. In particular, at least 12 high-risk genotypes (HR-HPV) are associated with the onset of cervical carcinoma and other cancers such as carcinomas of the anus, vagina, vulva, penis, oropharynx, nasopharynx, and oral cavity [11,12,13,14].

Aside from the full-blown role taken by HPV in the etiopathogenesis of malignant diseases, the detection of HPV DNA in semen, as well as in the ductus deferens, epididymis, testis, urethra, and external genitalia [15,16,17,18,19,20], has sparked some interest in the possible influence that male genital infection could have on semen quality, reproductive health, and infertility [21]. 

In particular, studies have highlighted how HPV could be linked with lower spontaneous pregnancy rates [22], poor reproductive outcomes, and higher rates of miscarriages in assisted reproductive technologies (ART) [23,24,25,26,27]. However, thus far, results are mainly conflicting and unclear.

The negative effect of HPV infection on basic sperm parameters and the underlying mechanism of such impairment have been investigated in several studies [28,29,30,31,32]. 

A possible correlation was observed between HPV sperm infection and cases of unexplained asthenozoospermia; but the literature data are still controversial and, in any case, the mechanism by which HPV virions could influence sperm motility is still unclear [25,33,34]. In some instances, anti-sperm antibodies (ASA), whose mean percentage is significantly higher in HPV-positive patients than in their negative counterparts, have been identified as the cause of alterations in sperm motility and concentration [19,35,36]. 

Another hypothesis identifies spermatozoa as carriers of HPV virions: it was shown that the L1 protein of the viral capsid can efficiently bind to syndecan-1, located on two sites of the sperm head equatorial region [25,34,37]. This tight association could cause impairment of the acrosomal reaction and embryonic development [23,24,25,26,34]. In fact, the eventual integration of viral DNA in the host genome may cause DNA damage, while the transferring of HPV DNA into the oocyte, with the subsequent activation of viral gene expression in the cells of the trophoblast and blastocyst, could be associated with negative effects on early embryo development [38]. 

Other authors suggested that an elevated reactive oxygen species’ (ROS) activity in HPV-positive seminal plasma could be linked to sperm membrane deterioration and, hence, to the exposition of sperm DNA to oxidative damage [19].

To date, few studies have investigated DNA fragmentation in HPV-infected semen samples. Some authors observed an increase in DFI in HPV-positive patients, whereas other authors failed to find a correlation [39,40,41,42,43,44]. 

The reason behind such discordant data could be a different localization of HPV virions in the urogenital male apparatus and in the elements that compose seminal fluid. In fact, the exact localization and origin of viral particles are still not clear: they could be located inside the sperm cell, in exfoliated urothelial epithelial cells, or in the prostatic fluid or, again, they can derive from partners or originate from non-dividing squamous epithelial cells that release the virions by an infectious virion-producing pathway [45,46,47]. 

Whatever the cause, HPV’s ability to infect spermatozoa and negatively affect their genomic integrity gains particular importance in assisted reproductive technology, where any natural selection of HPV-carrying sperm does not occur [34].

To shed light on the controversial impact of HPV infection on IVF it is mandatory to carefully analyze the alteration of sperm parameters and DNA integrity. In this respect, we performed a retrospective observational study on semen samples of 117 male partners of HPV-infected women attending IVF procedures. For each sample, seminal parameters were evaluated, HPV was detected by conventional protocol, and a sperm chromatin dispersion (SCD) test was carried out to assess spermatozoa DFI.

## 2. Materials and Methods

### 2.1. Patients and Sample Collection

A retrospective observational study was performed on a sample of 117 male partners of HPV-positive women, with a mean age of 38.6 ± 8.1 (SD) years. Semen samples were collected at “D’Arena”, Clinical Analysis and Diagnostics Laboratory, Vallo della Lucania, Salerno, by masturbation after a recommended sexual abstinence period of 3–5 days. After liquefaction at room temperature, sperm concentration, motility, and normal morphology were assessed according to World Health Organization guidelines for semen analyses [5]. An aliquot of the total semen of each patient was sent to the Virology Laboratory of UOC of Microbiology and Virology, Polyclinic Hospital, Palermo, Italy, for HPV detection, and another one was sent to the “Check Up” Polydiagnostic and Research Centre, Salerno, for DNA fragmentation analysis. 

Two groups of patients were defined: an HPV-negative group composed of 70 patients and an HPV-positive group composed of 47 patients. The inclusion criteria were as follows: no previous known genital infections; no current infection of *C. trachomatis*, *N. gonorrhoeae*, *U. parvum*, *U. urealyticum*, *M. hominis*, and *M. genitalium*; no seropositivity for human immunodeficiency virus type 1 or 2, human T-cell lymphotropic virus type 1 or 2, hepatitis B or C virus, and *T. pallidum*; no genetic disease such as cystic fibrosis; and no inflammatory disorders such as varicoceles. The study was performed according to The Code of Ethics of the World Medical Association (Declaration of Helsinki) [48].

Informed consent was obtained from all subjects. No funding or financial compensation was provided to the participants.

### 2.2. DNA Extraction

DNA was extracted from samples using the Qiamp DNA mini kit (Qiagen, Hilden, Germany). After liquefaction at room temperature, following the manufacturer’s protocol, 200 μL of semen samples were processed and elution was performed in 200 μL. The isolated DNA was quantified and analyzed with the spectrophotometer to determine its concentration and purity.

### 2.3. Detection and Type Identification of HPVs

After DNA extraction, both HPVs’ genome detection and genotyping were carried out using AMPLIQUALITY HPV-TYPE EXPRESS v3.0 (AB Analitica, Padua, Italy), which is based on the combined use of a PCR and a reverse hybridization assay. The amplification was performed according to the manufacturer’s instructions. After PCR, denatured amplicons were hybridized with specific probes for 40 HPV genotypes. The assay covered all currently known HR-HPV genotypes and probable HR-HPV genotypes (HPV-16, HPV-18, HPV-26, HPV-31, HPV-33, HPV-35, HPV-39, HPV-45, HPV-51, HPV-52, HPV-53, HPV-56, HPV-58, HPV-59, HPV-64, HPV-66, HPV-67, HPV-68 (68a and 68b), HPV-69, HPV-70, HPV-73, HPV-82) as well as several low-risk types (LR-HPV) (HPV-6, HPV-11, HPV-40, HPV-42, HPV-43, HPV-44, HPV-54, HPV-55, HPV-61, HPV-62, HPV-71, HPV-72, HPV-81, HPV-83, HPV-84, HPV-87, HPV-89, HPV-90). The distinction between high-risk and low-risk genotypes is based on the International Agency for Research on Cancer (IARC) classification [49].

### 2.4. Sperm DNA Fragmentation Analysis by SCD Test

Following the assessment of conventional semen parameters, samples were diluted in phosphate buffered saline solution 1× pH 7.4 (PBS Gibco, Invitrogen) at the concentration of 5–10 × 10^6^/mL to prevent sperm DNA halos from overlapping. If the sperm concentration was lower than 10 × 10^6^/mL, dilution was not performed. 

**Figure 1:**
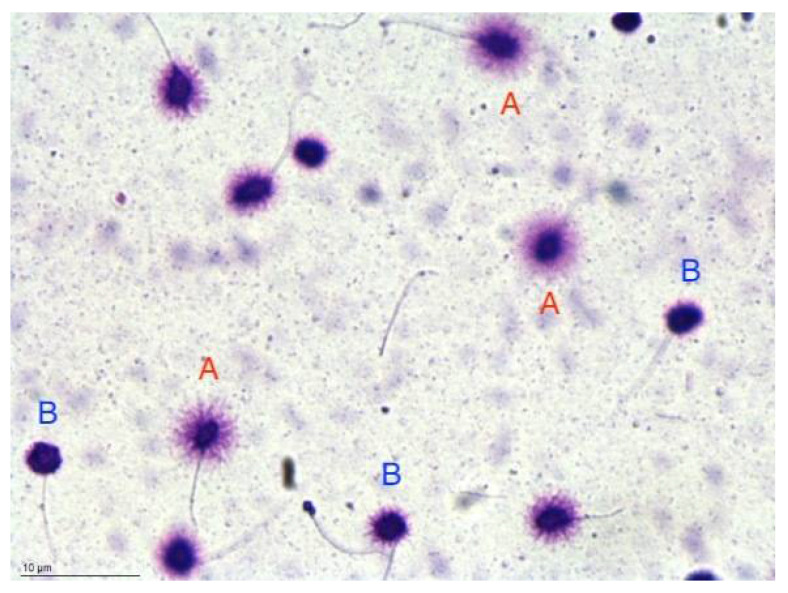
Interpretation of the halo sperm test. Normal sperms are indicated as A. Sperms with fragmented DNA are indicated as B. Bars: 10 μm.

Analysis of sperm DNA fragmentation was carried out using the technique described by Fernandez et al. [50] and Bosco et al. [51] using a brightfield microscopy Halosperm^®^ kit (Halotech DNA SL Spain; Figure 1). Briefly, a total of 500 sperms for each sample were observed and classified according to the size of the sperm DNA dispersion halo. Five different sperm DNA halo patterns were observed: big and medium, classified as not fragmented DNA, and small, absent halos, and degraded sperms, classified as fragmented. The values of the DNA fragmentation index (DFI) were evaluated as the percentage of fragmented nuclei relative to total cells. Figure 1 shows the interpretation of the sperm chromatin dispersion test (SCD) and was acquired through a Gigabit Camera (Basler Ace ACA780-75GC) connected to a brightfield microscopy (Nikon Ci-L).

### 2.5. Statistical Analysis

Data are presented as numbers and percentages for categorical variables, and continuous data are expressed as mean ± standard deviation (SD) or median and interquartile interval (IRQ = (Q1, Q3)). 

A test for normal distribution was performed by the Shapiro–Wilk test. A *t*-test was used to compare the mean of unpaired samples. When the distribution of samples was not normal, the Mann–Whitney test was used. Differences between groups were analyzed using the chi-square test or Fisher’s exact test for categorical variables. All tests with *p* < 0.05 were considered significant. All data were analyzed with Matlab statistical toolbox version 2008 (MathWorks, Natick, MA, USA) for 32-bit Windows.

## 3. Results

### 3.1. Seminal Parameters: HPV-Negative vs. HPV-Positive Samples

Of the 117 semen samples from male partners of HPV-positive women of infertile couples, 70 were HPV negative (59.8%) and 47 (40.2%) were HPV positive. Patients with HPV semen infection had a mean age of 38.6 ± 8.1 (SD) years, not different from the non-infected subjects. 

Table 1 shows no HPV-related effect on DFI (*p*-value = 0.32) and no significative differences between the HPV-negative vs. HPV-positive groups in seminal parameters: sperm concentration, total sperm number, and both progressive and total motility. We also observed a significantly lower rate of normal forms in HPV-positive samples in comparison to HPV-negative samples (*p* < 0.0001). In addition, HPV positivity was significantly associated with head defects (*p* = 0.0047), neck and midpiece defects (*p* = 0.0002), and tail defects (*p* = 0.0033). 

The relationship between HPV and its negative effect on sperm morphology remains weakly understood, but it could be due to the presence of HPV virions located in the different semen fractions.

### 3.2. Seminal Sample’s Parameter Compared with Lower Reference Limits (Fifth Centiles WHO 2010)

**Figure 2:**
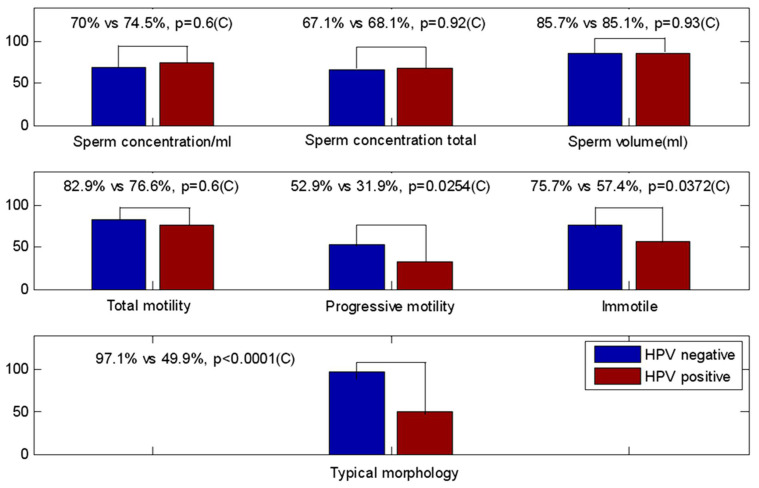
Percentages of patients with normal score for HPV-positive and -negative groups compared with lower reference limits (fifth centiles and their 95% confidence intervals) for semen characteristics. The asterisk was used for significant tests.

Figure 2 graphically depicts the percentages of patients with normal scores for semen characteristics, in HPV-positive and -negative groups, compared with lower reference limits (fifth centiles and their 95% confidence intervals) [5].

We observed statistically significant differences in progressive motility (*p* = 0.0254), immotile spermatozoa (*p* = 0.0372), and normal forms (*p* < 0.0001). 

This was an additional statistical analysis performed by comparing not parametric statistical indices, such as means or medians, but the percentages of patients with normal values, according to WHO 2010, between the two groups. This approach better highlighted the differences between seminal parameters and allowed us to point out how HPV-positive patients most frequently have seminal parameters of motility and morphology below the minimum reference limits.

### 3.3. Seminal Parameters of HPV-Positive Patients Stratified in Low and High Risk

Table 2 identifies variations in seminal parameters between low- and high-risk genotypes’ infected samples; a further statistical analysis was performed only on HPV-positive patients. No differences were observed except for DFI (*p* = 0.0283), which showed a significant increase in the high-risk group.

On the contrary, progressive motility and morphology, which varied in relation to HPV positivity, did not show any genotype-related alteration. This data confirmed that the viral presence alone, regardless of the type, produced alterations in the aforementioned parameters.

## 4. Discussion

Millions of people worldwide encounter difficulties at the time of conceiving; in about half of the pregnancy-seeking couples, male infertility is recognized as a determining factor [52]. 

To understand the causes underlying this condition, clinical practice involves the analysis of general quality semen parameters, such as concentration, count, viability, motility, and morphology. However, evidence suggests that the abnormalities of the mentioned elements are not fully indicative of negative fertility outcomes in populations of first-pregnancy planners, especially when assisted reproduction procedures are involved. On the contrary, DFI, an indicator of sperm DNA damage, has been strongly correlated with male infertility issues [6,7] (pp. 87–100). 

In the past years, ever-increasing attention has been paid to understanding how human papillomavirus male infection could influence semen impairment and negative pregnancy outcomes; but, until now, data were scarce and conflicting. In this context, this study aimed to examine the details of such a controversial correlation. 

The analyzed cohort of patients undergoing IVF procedures showed an HPV positivity rate of 40.2%. The discrepancy between this figure and recent meta-analytical data estimating a rate of seminal HPV positivity of 8.2% in the general population, and 20.9% among infertile men, is explained by our selection of only partners of HPV-positive women [53]. Among HPV-positive patients, HR genotypes were the most represented, as described by Sepulveda et al. [53], who described rates of 11.9% for HR-HPVs and 7.2% for LR-HPVs among infertile men.

The assessment of conventional seminal parameters, sperm concentration, motility, and semen volume, showed no significant differences between HPV-positive and -negative samples. These findings are added to what has already been described by several authors, such as Schillaci et al. [34], Golob et al. [30], and Luttmer et al. [47], who did not manage to find any association between HPV and semen abnormalities in males incapable of conceiving. Similarly, Rintala et al. [54] could not detect altered values of sperm concentration, vitality, and motility in HPV-positive semen of fertile fathers-to-be. 

In particular, by comparing the percentages of both HPV-positive and -negative patients with normal values identified according to the WHO 2010 classification, we more frequently observed in HPV-positive patients low progressive motility scores and high immotile spermatozoa percentages. Several studies are in accordance with these findings. In fact, Foresta et al. [55], Piroozmand et al. [36], Kato et al. [19], Moghini et al. [56], Boeri et al. [43], and Yang et al. [57] reported deleterious effects of HPV infection on sperm quality, finding motility as the most frequently affected parameter. An opposite correlation was observed in in vitro studies by Brossfield et al. [37] and Connelly et al. [39], which showed higher percentages of progressive and total motility in spermatozoa transfected with exogenous HPV DNA than untreated controls. 

As regards morphological parameters, the decrease in the normal morphology rate observed in HPV-positive samples was coherent with some of the literature data, namely, that of Piroozmand et al. [36], Moghimi et al. [56], and Yang et al. [57], which described substantial morphology abnormalities in the HPV-positive semen of both fertile [57] and infertile patients [36,56,57]. The HPV-induced morphology and progressive motility impairment found in our study are difficult to explain, but we can hypothesize that the mere presence of the virions, whatever fraction of the semen they are located in, could negatively influence these parameters. 

Sperm genomic damage, defined as the presence of chromosome rupture in the form of single- and double-strand DNA breaks, is strongly correlated with male infertility, difficulty of conception, and high rates of pregnancy loss [7] (pp. 87–100). Even though cutoffs are not yet included in WHO guidelines, the literature data consider a DFI < 20% as physiological and a value higher than 30% as indicative of infertility [58]. However, the evaluation of this parameter is recommended only in certain conditions, such as when environmental or pathological risk factors are present, after the failing of ART procedures, and in couples with unexplained infertility or recurrent pregnancy loss [7] (pp. 119–142). The clinical importance of evaluating DFI values when executing ART has been recently highlighted since a high DFI has been linked to poor clinical outcomes in ART cycles. The most likely hypothesis explaining these data indicates the incorporation of damaged sperm DNA into the embryonic genome as the cause of higher miscarriage rates and pregnancy loss [59,60,61].

The main triggers of a high DFI have been recognized in oxidative stress and in the induction of apoptotic processes, both triggered by aging, environmental factors (e.g., smoking, alcohol, drugs, diet, radiation, pollutants), testicular conditions (e.g., varicocele), and pathological status (e.g., infections, diabetes).

Sexually transmitted pathogens, specifically Chlamydia trachomatis and Mycoplasma, as well as hepatitis B antigens, have been indicated as agents of spermatozoa DNA fragmentation [62,63,64]. On the contrary, the opinion regarding human papillomavirus’ impact on DFI is not unanimous and research on the topic is quite limited. Current literature includes both studies that report a detrimental effect of HPV infection on the integrity of sperm DNA [39,43,53] and studies that have failed to find any association [41,42].

Gutiérrez et al. [42] and Kaspersen et al. [41] revealed no correlation between semen HPV DNA detection and a DFI value higher than 30%, while meta-analytic evidence [53] suggests that HPV positivity causes a significant detriment in DNA integrity. 

Results from in vitro studies showed the effects of HPV on DFI. Specifically, Connelly et al. [39] assessed how the exposition of spermatozoa to HPV-18 and -31 E6–E7 deoxyribonucleic acid fragments compromised sperm DNA integrity, while Lee et al. [40] described apoptotic fragmentation of p53 exons 5 and 8 after sperm exposition to HPV-16 and -18 virions.

Our investigation pointed out how HPV positivity does not modify DFI rates, except when comparing HR and LR genotypes. In fact, we found how infections with at least one HR-HPV showed a substantial increase in genomic damage compared to LR-only infections. As far as we know, hardly any study has analyzed the specific influence of HR- or LR-HPV on semen quality and DNA integrity. In particular, Boeri et al. [43] described how a substantial genomic impairment was univariably associated with HR-HPV infection in a cohort of men affected by primary infertility.

These findings indicate that it would be appropriate, especially for LR types, to consider the heterogeneity of HPV infections regarding the exact localization of viral particles. Indeed, at the beginning of the infection, HPV virions that originate from the partner are probably mainly localized in seminal plasma and have a limited effect on spermatozoa. Then, when the HPV virions are produced by the infected epithelial cells, they start to be detected also in the spermatozoa fraction [46]. Virions could have a major detrimental effect on semen at this stage. Another often overlooked element is the virion-to-spermatozoa ratio, which can be important to highlight a detrimental effect on sperm DNA damage. The majority of the cited papers studying the viral influence on semen quality do not evaluate the exact localization of HPV viral particles in seminal components or the mentioned ratio.

Therefore, we propose to use a protocol that can easily reveal the presence of viral particles in the different fractions that compose seminal fluid. 

In our previous study [65], we highlighted the heterogeneity of HPV infections in semen, even when multiple genotype infections occur. For the first time, we applied a differential lysis protocol to purify DNA from each fraction of semen (plasma, somatic cells, and spermatozoa) separately. It was revealed that all semen components (spermatozoa, somatic cells, plasma) can contain viral DNA, often in different amounts and sometimes only in the individual elements. 

This valid method, recently included in the ESHRE guideline for MAR (medically assisted reproduction) in patients with viral infection/disease [66] (p. 113), allows an easy distinction of different semen infections and their diverse impact on spermatozoa parameters. In particular, the location of the virions in the different sperm fractions tells something about the natural history of the infection and that the DFI may vary in time according to the stage of the infection. As DFI is exclusively evaluated into spermatozoa, it is advisable to verify in which semen fraction HPV virion is located, by a differential lysis procedure [34], before performing a DFI assay. 

Since it is possible to extract DNA from seminal plasma, sperms, and epithelial cells separately [45,65], in order to investigate whether the presence of HPV in semen affects the integrity of sperm DNA and fertility, we suggest verifying, only in LR-HPV, in which semen fraction HPV is located before performing DFI assays.

## 5. Conclusions

Based on our findings, we can conclude that HPV semen infection, regardless of HPV type and exact location of the virions, significantly impairs spermatozoa’s progressive motility, morphology, and immotile sperm rate. Conversely, the increased rate of DFI was a genotype-related alteration since it was only observed in HR-HPV-infected samples when compared to LR-HPV-infected ones. This is a crucial result as, to the best of our knowledge, the different influences of HR and LR genotypes on semen parameters and sperm genomic integrity have hardly been analyzed. Therefore, our work can explain the conflicting data reported in the literature about HPV and sperm DNA damage. 

Furthermore, since the effect of HPV infection on seminal parameters could be influenced by the exact localization of viral particles in different semen fractions, when it comes to LR-HPV infection, it would be advisable to perform the differential lysis protocol before testing the DFI. This approach would allow the identification of spermatozoa HPV infection, which could have the highest negative impact on genomic integrity and male fertility. 

## 6. Limitations

In this study, there were some results, such as the comparison between HPV-positive low- and high-risk patients, where the subgroups analyzed were relatively small, especially the low-risk group. Therefore, in this case, they should be interpreted as preliminary results and should encourage future research.

## Figures and Tables

**Table 1:** Seminal parameters of total patients and HPV-negative and HPV-positive groups.

Parameters	Total	HPV Negative	HPV Positive	HPV-Negative vs. Positive *p*-Value (Test)
Patients	117	70	47	
DFI%	28.7 ± 12.9	28.5 ± 14.3	28.9 ± 10.6	
	27.5 (20–35)	25.1 (19–33.75)	28.5 (23.65–35.5)	*p* = 0.32 (MW)
Sperm concentration/mL	45.0 ± 47.6	49.6 ± 51.9	38.3 ± 40.0	
	30 (13–58)	30 (8.5–70)	29 (14.5–52)	*p* = 0.41 (MW)
Total sperm number	120.7 ± 137.1	123.5 ± 144.9	116.5 ± 125.9	
	73.6 (26–153)	75 (23.5–149.8)	68.2 (30.85–157.5)	*p* = 0.95(MW)
Semen volume (mL)	2.9 ± 1.6	2.8 ± 1.6	3.0 ± 1.7	
	2.7 (1.8–3.5)	2.5 (1.7–3.2)	3 (2–4)	*p* = 0.36 (MW)
Progressive motility%	28.8 ± 15.3	29.6 ± 15.0	27.7 ± 15.8	
	30 (20–40)	35 (20–40)	25 (20–36.5)	*p* = 0.21 (MW)
Non-progressive%	16.2 ± 9.4	16.4 ± 10.4	16.0 ± 7.6	
	15 (10–20)	13.5 (10–20)	15 (10–20)	*p* = 0.68 (MW)
Total motility%	45.0 ± 16.4	46.0 ± 16.8	43.7 ± 15.9	
	50 (40–55)	50 (40.75–55)	45 (40–50)	*p* = 0.13 (MW)
Immotile%	54.2 ± 16.7	52.7 ± 17.2	56.3 ± 15.9	
	50 (45–60)	50 (45–56.5)	55 (50–60)	*p* = 0.08 (MW)
Normal forms%	6.8 ± 5.1	8.5 ± 5.4	4.4 ± 3.4	
	6 (2–10)	8 (4–12)	3 (2–6.5)	*p* < 0.0001 * (MW)
Head defects%	67.8 ± 16.9	64.1 ± 16.2	73.1 ± 16.7	*p* = 0.0047 * (T)
	67 (56–80)	65 (55–74)	71 (59.5–90)	
Neck and midpiece defects%	11.8 ± 8.5	9.2 ± 7.0	15.5 ± 9.1	
	10 (5–17)	8 (3–15)	15 (8.5–20)	*p* = 0.0002 * (MW)
Tail defects%	8.2 ± 8.5	6.1 ± 6.1	11.2 ± 10.5	
	5 (2–13.25)	4 (1–10)	10 (3.5–16)	*p* = 0.0033 * (MW)
Amorphous%	15.7 ± 8.0	15.4 ± 7.8	16.2 ± 8.2	
	15 (10–20)	15 (10–21)	15 (10–20)	*p* = 0.83 (MW)
HPV	40.2% (47)	—	—	—

* Significant test (*p* < 0.05); MW = Mann-Whitney test; T = *t*-Student test for normal distribution was performed by Shapiro–Wilk test; data are described by mean ± SD, median (IQR), or percentage.

**Table 2:** Seminal parameters of HPV-positive patients stratified in low and high risk.

Parameters	Low Risk	High Risk	*p*-Value (Test)
Patients	11	36	
DFI%	22.9 ± 8.7	30.8 ± 10.5	*p* = 0.0283 * (T)
	25 (16–28)	31 (25–37.5)	
Sperm concentration/mL	34.6 ± 26.0	39.4 ± 43.6	
	32 (18–53.5)	27 (13.75–50.5)	*p* = 0.78 (MW)
Total sperm number	114.1 ± 106.6	117.3 ± 132.6	
	68.2 (46.3–178.7)	69 (27.1–146.8)	*p* = 0.78 (MW)
Semen volume (mL)	3.0 ± 1.2	3.0 ± 1.8	
	3.1 (2.2–4)	2.7 (1.95–3.85)	*p* = 0.59 (MW)
Progressive motility%	29.6 ± 18.9	27.1 ± 15.0	*p* = 0.66 (T)
	35 (20–40)	25 (20–31.25)	
Non-progressive%	13.2 ± 9.3	16.9 ± 7.0	*p* = 0.16 (T)
	10 (10–15)	20 (10–20)	
Total motility%	42.7 ± 22.7	43.9 ± 13.6	
	50 (35–55)	45 (40–50)	*p* = 0.57 (MW)
Immotile%	57.3 ± 22.7	56.1 ± 13.6	
	50 (45–65)	55 (50–60)	*p* = 0.57 (MW)
Normal forms%	4.6 ± 3.3	4.4 ± 3.5	
	3 (2.5–6.5)	3.5 (2–6.3)	*p* = 0.75 (MW)
Head defects%	70.0 ± 16.1	74.0 ± 17.0	
	62 (59–80.5)	76 (61.3–90)	*p* = 0.55 (MW)
Neck and midpiece defects%	16.6 ± 6.7	15.2 ± 9.8	*p* = 0.68 (T)
	20 (12.5–20)	15 (7–20.5)	
Tail defects%	10.7 ± 9.8	11.3 ± 10.9	
	5 (4.5–17)	10 (3–15)	*p* = 0.92 (MW)
Amorphous%	16.2 ± 8.3	16.3 ± 8.3	
	15 (10–20)	15 (10.75–20)	*p* = 0.89 (MW)

* Significant test (*p* < 0.05); MW = Mann–Whitney test; T = *t*-Student test, test for normal distribution was performed by Shapiro–Wilk test. Data are described by mean ± SD, median (IQR), or percentage.

## Data Availability

Not applicable.
